# Parenthood and neurosurgery in Europe a white paper from the European Association of Neurosurgical Societies’ Diversity in Neurosurgery Committee Part I – Family Planning and Practice during Pregnancy

**DOI:** 10.1016/j.bas.2023.102690

**Published:** 2023-11-02

**Authors:** Pia Vayssiere, Marike Broekman, Claudio Cavallo, Doortje Engel, Uri Pinchas Hadelsberg, Gökce Hatipoglu Majernik, Anke Hoellig, Tijana Ilic, Claudia Janz, Hanne-Rinck Jeltema, Dorothee Mielke, Ana Rodríguez-Hernández, Yu-Mi Ryang, Saeed Fozia, Nikolaos Syrmos, Kristel Vanchaze, Silvia Hernandez-Duran

**Affiliations:** a Department of Neurosurgery, Hôpitaux Universitaires de Genève (HUG), Geneva, Switzerland; b Faculty of Medicine, Université de Genève (UNIGE), Geneva, Switzerland; c Dept of Neurosurgery, Haaglanden Medical Center, The Hague and Dept of Neurosurgery, Leiden University Medical Centre, Leiden, the Netherlands; d Department of Neurosurgery, Neurocenter of Southern Switzerland, Regional Hospital of Lugano, Lugano, Switzerland; e Labor Dr. Risch, Buchs, St. Gallen, Switzerland; f Department of Neurosurgery, Hadassah University Medical Center, Jerusalem, Israel; g Department of Clinical Neurological Sciences, Schulich School of Medicine and Dentistry, Western University, London, Ontario, Canada; h Department of Neurosurgery, University Hospital RWTH Aachen, Germany; i National Department of Neurosurgery, Centre Hospitalier de Luxembourg, Europe; j Städtisches Klinikum Solingen, Neurochirurgische Klinik, Gotenstrasse 1, 42563, Solingen, Germany; k Department of Neurosurgery, University Medical Center Groningen, Groningen, the Netherlands; l Department of Neurosurgery, University Hospital Göttingen, Göttingen, Germany; m Dept. of Neurological Surgery, Germans Trias i Pujol University Hospital, Universidad Autónoma, Barcelona, Spain; n Dept. of Neurosurgery &Center for Spinetherapy, Helios Klinikum Berlin-Buch, Germany; o Department of Neurosurgery at Leeds General Infirmary, Leeds, United Kingdom; p Aristotle Univesrity of Thessaloniki, Greece; q AZ St Lucas, Ghent and AZ Alma, Eeklo, Belgium; r Klinik für Neurochirurgie, Universitätsmedizin Göttingen, Robert-Koch-Straße 40, 37075, Göttingen, Germany

**Keywords:** Family planning, Pregnancy, Parenthood, Parental leave, Maternal leave

## Abstract

**Introduction:**

Family and work have immensely changed and become intertwined over the past half century for both men and women. Additionally, alongside to traditional family structures prevalent, other forms of families such as single parents, LGBTQ + parents, and bonus families are becoming more common. Previous studies have shown that surgical trainees regularly leave residency when considering becoming a parent due to the negative stigma associated with pregnancy during training, dissatisfaction with parental leave options, inadequate lactation and childcare support, and desire for greater mentorship on work-life integration. Indeed, parenthood is one of the factors contributing to attrition in surgical specialities, neurosurgery not being an exception.

**Research question:**

The Diversity in Neurosurgery Committee (DC) of the European Association of Neurosurgical Societies (EANS) recognizes the challenges individuals face in parenthood with neurosurgery and wishes to address them in this white paper.

**Materials and methods:**

In the following sections, the authors will focus on the issues pertaining to family planning and neurosurgical practice during pregnancy in itemized fashion based on an exhaustive literature search and will make recommendations to address the matters raised.

**Results:**

Potential solutions would be to further improve the work-family time ration as well as improving working conditions in the hospital.

**Discussion and conclusion:**

While many obstacles have been quoted in the literature pertaining to parenthood in medicine, and in neurosurgery specifically, initiatives can and should be undertaken to ensure not only retention of colleagues, but also to increase productivity and job satisfaction of those seeking to combine neurosurgery and a family life, regardless of their sexual identity and orientation.

## Highlights

• A recent study on European neurosurgeons revealed that men were significantly more likely to have children than their female counterparts in our specialty.
• Studies conducted in the United States have shown that female general surgeons have fewer children and choose to postpone pregnancy, while as many as 40% of them remain childless, as opposed to only 8% of their male counterparts at their same career level and age.
• Women thoracic surgeons begin their families later in life and have fewer children compared to other women of the same age and male colleagues at the same hierarchical level.
• Assisted reproductive techniques, adoption, or surrogacy are costly and lack strong workplace support in surgery, disproportionately impacting women and same-sex couples.

## 1 Introduction

Family and work have immensely changed and become intertwined over the past half century for both men and women. Balancing career and domestic life remain one of the biggest challenges of modern life (Modern Parenthood | Pew Research Center). Additionally, alongside to traditional family structures prevalent, other forms of families such as single parents, LGBTQ + parents, and bonus families are becoming more common in modern society (The Evolution of Families), (Itao and Kaneko, 2021). In highly demanding occupations, such as neurosurgery, the balance between work and family is notoriously more elusive and difficult to achieve (Modern Parenthood | Pew Research Center).

Previous studies have shown that surgical trainees regularly leave residency when considering becoming a parent due to the negative stigma associated with pregnancy during training, dissatisfaction with parental leave options, inadequate lactation and childcare support, and desire for greater mentorship on work-life integration (Rangel, 2018). The same holds true for specialists. Parenthood has also been shown to negatively influence the career path of women in particular, as female physicians are less advanced in their specialty qualification, are less prone to choosing prestigious surgical fields, have a mentor less often, and more often work at small hospitals or in private practice (Buddeberg-Fischer et al., 2010). On the other hand, there is a strong cultural support for mothers'-but not fathers' parental timely commitments, resulting in maternal caregiving, even when couples want to share parental leave (Beglaubter, 2017). Commonly, parents' (both mothers' and fathers') wages are also negatively affected when they decide to utilize parental leave (Kahn et al., 2014), (Database et al., 2019). Thus, parenthood is one of the factors contributing to attrition in surgical specialities, neurosurgery not being an exception.

The Diversity in Neurosurgery Committee (DC) of the European Association of Neurosurgical Societies (EANS) recognizes the challenges individuals face in parenthood with neurosurgery and wishes to address them in this white paper. In the following sections, the authors will focus on the issues pertaining to family planning and neurosurgical practice during pregnancy in itemized fashion based on an exhaustive literature search and will make recommendations to address the matters raised.

## 2 Deciding to have a family

Parenthood poses a conundrum even before the birth of a child itself. In our literature search, we identified several challenges faced by physicians planning to start a family, listed in the following subsections.

### 2.1 Family planning

• A recent study on European neurosurgeons revealed that men were significantly more likely to have children than their female counterparts in our specialty (Lambrianou et al., 2022).
• Studies conducted in the United States have shown that female general surgeons have fewer children and choose to postpone pregnancy, while as many as 40% of them remain childless, as opposed to only 8% of their male counterparts at their same career level and age (Stephens and Heisler, 2020).
• Women thoracic surgeons begin their families later in life and have fewer children compared to other women of the same age and male colleagues at the same hierarchical level (Pham et al., 2014).
• Assisted reproductive techniques, adoption, or surrogacy are costly and lack strong workplace support in surgery, disproportionately impacting women and same-sex couples (Atkinson et al., 2022).

### 2.2 Societal roles and stipulations

Even though great advances have been made in society in terms of gender equality, societal roles continue to place child rearing predominantly in the hands of women. For women in neurosurgery, this may pose an extra hurdle when deciding to start a family, for it may imply having to sacrifice time otherwise dedicated to their career in favor of their family goals.

• According to a continental survey, European women in neurosurgery take on more responsibilities at home, especially during the child-rearing years (Lambrianou et al., 2022).
• Women surgeons in the United States report that they need more time with their children, contrary to men surgeons (Mayer et al., 2001).
• Among married or partnered US-American physicians with children, after adjustment for work hours, spousal employment, and other factors, women spent 8.5 more hours per week on domestic activities. In the subgroup with spouses or domestic partners who were employed full-time, women were more likely to take time off during disruptions of usual childcare arrangements than men (Jolly et al. 2014).
• 26% of married male graduates list their wife as a homemaker, while all the married female surgeons in one study had a spouse working in the same or another profession (Mayer et al., 2001).
• Strong cultural support for mothers'-but not fathers'-time with baby has tipped the scales toward maternal caregiving, even when couples wanted to share parental leave (Beglaubter, 2017).
• While diverse families with members of the LGBTQ + community are commonplace in any workplace, legislation has not necessarily caught up with the times. Only 18 EU countries give LGBTQ + parents corresponding rights, mostly through second-parent adoption or a similar instrument. A smaller number of EU countries allow joint parenthood for same-sex (mostly lesbian) couples from birth. Same-sex couples are not entitled to the same rights as heterosexual couples when it comes to parenthood in most European countries (Table 1). Same-sex couples in Europe also report emotional conflicts driven by introjected heteronormative assumptions about family. This results in these individuals being confronted with many hurdles when deciding to become parents (Picken and Janta, 2019), (“Even where countries in Europe).

**Table 1:** European countries where joint legal parenthood for same-sex couples is possible and associated parental leave policies (Picken and Janta, 2019).

Joint legal parenthood for same-sex couples possible	Austria
	Belgium
	Denmark
	Estonia
	Finland
	France
	Germany
	Ireland
	Italy
	Luxembourg
	Malta
	The Netherlands
	Portugal
	Spain
	Sweden
	Slovenia
	United Kingdom
Joint legal parenthood for same-sex couples NOT possible	Bulgaria
	Croatia
	Czechia
	Cyprus
	Greece
	Hungary
	Latvia
	Lithuania
	Poland
	Romania
	Slovakia
All types of partners of parents can take parental leave	Austria
	Belgium
	Estonia
	Finland
	France
	Germany
	Ireland
	Luxembourg
	The Netherlands
	United Kingdom
Only legal parents can take parental leave	Bulgaria
	Czechia
	Greece
	Italy
	Latvia
	Lithuania
	Portugal
	Romania
	Spain
Only married or registered partners can take parental leave	Hungary

### 2.3 Access to parental leave

• To begin with, access to parental leave is flawed even within heterosexual couples. This leave is not a mere one-time event in life (i.e. pregnancy or the period after childbirth). Indeed, it is noticed several years forward, when the parent is not able to participate in pick-ups from school/kindergarten and throughout a neurosurgeon's career, more so affecting female neurosurgeons and contributing to the negative effect on training and performance in our field.
• For members of the LGBTQ + community, access to parental leave equal to heterosexual couples is not possible in most European countries (Table 1).

### 2.4 Stigma and worries about career advancement

• In European neurosurgery, 72% of female neurosurgeons reported worrying that their career prospects could be negatively affected by their desire to have children (Wolfert et al., 2019).
• 25% of department chairs in Japan are reluctant to hiring female neurosurgeons because of childbearing/rearing issues (Fujimaki et al., 2016).
• In US-American cardiothoracic surgery, 61% of women vs 16% of men felt pregnancy would be viewed unfavorably among peers (Pham et al., 2014).
• In US-American general surgery, the overall magnitude of perceived negative attitudes is greater among male peers than female peers and among faculty than peers; even women residents hold negative views of pregnancy among their colleagues during training. More than half of all women surgeons delay childbearing until they are in independent practice, post-training. Surgical residents and faculty of both sexes exerted negative influences with regard to consideration of childbearing (Turner et al., 2012).
• Physician pregnancy impacted colleagues through perceived increased workload and resulted in persistent stigmatization and discrimination despite work productivity and academic metrics being independent of pregnancy events (Casilla-Lennon et al., 2022).
• Academic program directors perceive that becoming a parent negatively affects females' performance more than males' (Acai et al., 2018), (Sandler et al., 2016).
• In several European countries, surgical practice during pregnancy is prohibited; this, in turn, might negatively influence a woman's desire and plans for a family and/or her career (Fritze-Büttner et al., 2020).

### 2.5 Potential solutions

• Leading by example.
  o Having a woman surgeon leader in a team radically changes the perception of being a woman in surgery. Female surgical residents with a female program director felt supported to consider pregnancy during training as opposed to those with a male program director (Mundschenk et al., 2016).
  o The most cited quality for an ideal mentor among gynecology residents and fellows was the ability to balance family and full-time practice (Gordinier et al., 2000).
    ⁃ Showcasing success stories of neurosurgeons who have achieved professional leadership while having a family through the EANS DC online repository and social media can generate positive role models for compatibility between neurosurgery and parenthood and empower those considering having a family but are afraid to do so because of the aforementioned factors.
• Transparent communication.
  oPromotion of clear family leave policies for trainees and practitioners, even before pregnancy has ensued (Gupta et al., 2020).
  oEncouragement of forethought and flexibility to tackle obstacles inherent to pregnancy and the early stages of child rearing (Gupta et al., 2020).
  oFamily-friendly policies can improve worker retention, wellbeing, productivity, and performance, as well as job satisfaction and loyalty (H. F. O. R. Business).
  oAdherence to national regulations pertaining to parental leave can ease the decision-making process of aspiring parents in neurosurgery. European parental leave policies are summarized in Table 2 (Database et al., 2019), (van Belle, 2016). Nevertheless, departments can make accommodations for families, in particular in cases of LGBTQ + families.
    ⁃ An online repository of parental leave policies within neurosurgical departments across Europe could help aspiring parents identify those centers that would be most compatible with their personal/family goals.

**Table 2:** Parental leave policies in Europe.

Country	Paid maternity leave (weeks)	Payment (percentage of salary)	Paid paternity leave (weeks)	Payment (percentage of salary)	Paid parental leave to mothers (weeks)	Payment (percentage of salary)	Paid parental leave for fathers (weeks)	Payment (percentage of salary)
Austria	16	100	0	0	44	76	9	76
Belgium	15	64	2	73	17	20	17	20
Bulgaria	59	90	2	90	52	33	0	0
Croatia	30	100	0	0	26	42	0	0
Cyprus	18	75	2	75	0	0	0	0
Czech Republic	28	61	1	61	35	84	1	61
Denmark	18	53	2	53	32	53	0	0
Estonia	20	100	2	100	146	44	0	0
Finland	18	74	3	63	144	19	6	63
France	16	90	2	90	26	14	26	14
Germany	14	100	0	0	44	65	9	65
Greece	43	50	1	100	0	0	0	0
Hungary	24	70	1	100	136	38	0	0
Ireland	26	27	2	27	0	0	0	0
Israel	15	100	0	0	0	0	0	0
Italy	22	80	1	100	26	30	0	0
Latvia	16	80	1	100	78	50	0	0
Lithuania	18	100	4	100	44	100	0	0
Luxembourg	20	100	2	100	17	67	17	67
Malta	18	86	1	100	0	0	0	0
Netherlands	16	100	1	100	0	0	0	0
Norway	13	94	0	0	78	40	10	94
Poland	20	100	2	100	32	68	0	0
Portugal	6	100	5	100	24	60	17	44
Romania	18	85	1	100	91	85	4	85
Slovakia	34	75	0	0	130	21	0	0
Slovenia	15	100	4	90	37	90	0	0
Spain	16	100	4	100	0	0	0	0
Sweden	13	78	1	59	43	57	13	78
Switzerland	14	59	0	0	0	0	0	0
Turkey	16	67	1	100	0	0	0	0
UK	39	30	2	19	0	0	0	0

Definitions: Maternity leave (or pregnancy leave): employment-protected leave of absence for employed women directly around the time of childbirth (or, in some countries, adoption). Paternity leave: employment-protected leave of absence for employed fathers at or in the first few months after childbirth. Paternity leave is not stipulated by international convention. Parental leave: employment-protected leave of absence for employed parents, which is often supplementary to specific maternity and paternity leave periods, and frequently, but not in all supplementary to specific maternity and paternity leave periods, and frequently, but not in all countries, follows the period of maternity leave.

## 3 Neurosurgical practice and pregnancy

While planning a family already poses a myriad of challenges, neurosurgical practice during pregnancy in Europe can be hindered by departmental regulations concerned with the safety for both the mother and the fetus. In the following sections, we provide the evidence regarding potential occupational hazards for pregnant women practicing neurosurgery.

### 3.1 Occupational hazards

A study conducted in the United States showed that female surgeons operating 12 or more hours per week during the last trimester of pregnancy are at higher risk of major pregnancy complications compared with those operating less than 12 h per week (OR, 1.57; 95% CI, 1.08–2.26) (Rangel et al., 2021). This is due to a combination of very heterogeneous factors. The most important, modifiable occupational hazards are summarized below; this, however, is by no means an exhaustive list.

• Radiation.
  oThe Council Directive 2013/59/EURATOM of December 5, 2013 stipulates that Member States shall ensure that the protection of the unborn child is comparable with that provided for members of the public. Thus, as soon as a pregnant worker informs the employer of the pregnancy, in accordance with national legislation, the employer, shall ensure that the equivalent dose to the unborn child is as low as reasonably achievable and unlikely to exceed 1 mSv during at least the remainder of the pregnancy (European Society of Radiology (ESR) et al., 2015).
• Anesthetics.
  oWaste anesthetic gases (WAG) are not only a proven occupational healthcare hazard for anesthesiologists, but for any employee present in the operating room, recovery unit and intensive care unit (Gaya da Costa et al., 2021).
  oHigher rates of spontaneous abortion, teratogenic effects, cancer and hepatic and renal diseases were found in female workers (Occupational Disease among Operating Room, 1974).
  oGenomic instability, cytotoxicity and proliferative risks were seen in samples of anaesthesiologists compared to non-exposed internal medicine colleagues (Çakmak et al., 2019).

### 3.2 Potential solutions

• Personal protective equipment (PPE) and digital dosimeters can enable those pregnant surgeons to continue their practice safely.
  oPPE (lead aprons) and a digital or extra dosimeter in order to protect the unborn child, even in the case of working in interventional radiology (Vu and Elder, 2013).
  oConsidering that a pedicle screw placement leads to an undetectable exposure of the body protected by a lead apron (Mroz et al., 2011), there is no conclusive evidence to prevent pregnant women from carrying out such surgical procedures, provided PPE is available. Women can also use aprons specifically designed for pregnancy with extra inserts over the pelvis leading up to 1 mm lead equivalent protection (Nickoloff et al., 1999).
• Total intravenous anesthesia is healthier for both patients and staff, but much more expensive in most studies/countries (Occupational Disease among Operating Room, 1974), (Çakmak et al., 2019), (Kaur et al., 2018). Implementing a scavenging system that sucks away 40–90% of the waste anesthetic gases is more cost-effective, but the concentrations measured of WAGs is still above the recommended limit. With a high percentage of staff in their child-baring years it is a political decision of an institution or healthcare system to act or not.
• Departmental standards of practice (SOP) regarding the duties of pregnant women and policies regarding maternal and fetal safety during pregnancy can provide clarity in communication and facilitate both the integration of pregnant women into the regular neurosurgical workforce and their professional success. By informing and guiding surgeons (and other operating theatre staff) hazards can be manageable, leading to a healthy working environment and minimalization of work absence before and during motherhood.

## 4 Conclusions

While many obstacles have been quoted in the literature pertaining to parenthood in medicine, and in neurosurgery specifically, initiatives can and should be undertaken to ensure not only retention of colleagues, but also to increase productivity and job satisfaction of those seeking to combine neurosurgery and a family life, regardless of their sexual identity and orientation.

## Methodology

For composing these white papers, the European Association of Neurosurgical Societies (EANS)'s Diversity Committee (DC) recruited neurosurgeon volunteers from all member countries, including parents, aspiring parents, and individuals without any desire to have a family to create a diverse and representative working group (WG).

Meetings were organized via the videoconference platform Zoom® (Zoom Video Communications, Inc. California, USA) on a bimonthly basis for a one-year period. During the initial phase of the project, meetings were carried out on a biweekly basis and different aspects of parenthood were discussed, taking into consideration the personal experiences of the WG members. During these initial discussions, obstacles and challenges in practicing neurosurgery while becoming and/or being a parent were recognized.

In a second stage, literature pertaining to the obstacles and challenges identified was collected. For the literature search, PubMed and Google Scholar were utilized to identify scientific papers pertaining to parenthood in medicine. Furthermore, European agencies were digitally consulted to clearly define the legal framework within which parents and aspiring parents are to practice neurosurgery, including parental leave policies and parental leave rights in Europe. This information was then summarized and compiled as a GoogleDoc (Google LLC California, USA).

Once literature was collected and sorted according to the pertaining problems identified, WG members discussed potential, implementable solutions to tackle points discussed. Then, a second round of literature searches was conducted to identify evidence supporting potential solutions. To ensure objective interpretation and collection of data, every member of the WG reviewed the literature identified, following the principles of horizontal organizations.

Finally, manuscripts were drafted and reviewed by all the members of the WG.

## Declaration of competing interest

All the authors who have participated in the drafting, writing and submitting this manuscript report no conflict of interests pertaining to this paper.
